# Insights into the Role of Yeast eIF2A in IRES-Mediated Translation

**DOI:** 10.1371/journal.pone.0024492

**Published:** 2011-09-07

**Authors:** Lucas C. Reineke, Yu Cao, Diane Baus, Nasheed M. Hossain, William C. Merrick

**Affiliations:** 1 Department of Biochemistry, Case Western Reserve University, School of Medicine, Cleveland, Ohio, United States of America; 2 Department of Molecular Virology and Microbiology, Baylor College of Medicine, Houston, Texas, United States of America; 3 Department of Engineering Management, Information and Systems, Southern Methodist University, Dallas, Texas, United States of America; 4 The Department of Medicine, University of Chicago Medical Center, Chicago, Illinois, United States of America; Victor Chang Cardiac Research Institute (VCCRI), Australia

## Abstract

Eukaryotic initiation factor 2A is a single polypeptide that acts to negatively regulate IRES-mediated translation during normal cellular conditions. We have found that eIF2A (encoded by *YGR054w)* abundance is reduced at both the mRNA and protein level during 6% ethanol stress (or 37°C heat shock) under conditions that mimic the diauxic shift in the yeast *Saccharomyces cerevisiae*. Furthermore, eIF2A protein is posttranslationally modified during ethanol stress. Unlike ethanol and heat shock stress, H_2_O_2_ and sorbitol treatment induce the loss of eIF2A mRNA, but not protein and without protein modification. To investigate the mechanism of eIF2A function we employed immunoprecipitation-mass spectrometry and identified an interaction between eIF2A and eEF1A. The interaction between eIF2A and eEF1A increases during ethanol stress, which correlates with an increase in IRES-mediated translation from the *URE2* IRES element. These data suggest that eIF2A acts as a switch to regulate IRES-mediated translation, and eEF1A may be an important mediator of translational activation during ethanol stress.

## Introduction

Originally eukaryotic initiation factor 2A (eIF2A), a single polypeptide, was purified based on its ability to direct binding of initiator methionyl-tRNA (met-tRNA_i_) to the 40S ribosome in an AUG-dependent manner and its ability to catalyze poly(U)-directed polyphenylalanine synthesis at low [Mg^+2^] [1]. Eukaryotic initiation factor 2 (eIF2), a heterotrimeric protein complex, was also found to promote binding of met-tRNA_i_ to the 40S ribosome, but in a GTP-dependent manner [2]. However, comparative analysis indicated that eIF2A was less efficient at met-tRNA_i_ delivery to the 40S ribosomal subunit on artificial templates, and was inactive using globin mRNA as a template for polypetide synthesis [3]. This initial work established the idea of competition between two distinct pathways for delivery of methionyl-tRNA to the 40S ribosomal subunit during translation initiation. However, research on the role of eIF2A in translational control ceased for 25 years because of the absence of any apparent activity on a native transcript [3]. Identification of a yeast homolog to eIF2A (corresponding to yeast gene *YGR054w)* reignited efforts to characterize eIF2A because of the potential for genetic dissection of the pathway for eIF2A-mediated regulation of translation [4].

Zoll *et al.* found that yeast and human eukaryotic initiation factor 2A (eIF2A; *YGR054W* in yeast) are 28% identical and 58% homologous, which suggests a conserved function throughout evolution [4]. Since the identification of a yeast homolog of eIF2A, much work has been done to identify the biological and physical properties of the protein. Yeast eIF2A has been shown to localize to 40S and 80S ribosomes, consistent with its role in translation initiation [5]. Eukaryotic initiation factor 2A has been shown to specifically repress translation of the *URE2* internal ribosome entry site without affecting cap-dependent translation (IRES; [5]). Translation of two other yeast IRES elements, *GIC1* and *PAB1*, were also repressed by eIF2A [6].

To further characterize eIF2A protein, we have employed several approaches. First, we investigated the response of eIF2A protein and mRNA to several stresses since eIF2A regulates expression of IRES-containing mRNAs and has little effect on “standard” mRNA translation [3], [5], [6]. We reasoned that eIF2A protein would respond in one of two ways to stress: i. eIF2A protein would be turned over to promote translation of IRES-containing mRNAs, or ii. eIF2A protein would be modified to enhance initiator methionyl-tRNA delivery to the ribosome during the stress conditions. Second, we investigated proteins that may be important for the eIF2A pathway of translation by conducting immunoprecipitation/mass spectrometry experiments. These approaches led to a model in which eIF2 and eIF2A compete for delivery of initiator methionyl-tRNA to the 40S subunit and modulation of eIF2A activity allows discrimination between the two pathways.

## Methods

### A. Plasmids and cloning

The yeast plasmid YCplac111-YP was constructed by inserting the natural yeast eIF2A promoter located within the 800 bp upstream of the AUG codon into YCplac111galp (originally reported in [4]) to replace the *GAL1* promoter. This plasmid was used in all stress experiments unless otherwise indicated and in the initial screen for eIF2A-interacting partners with pTB328, the parent vector for YCplac111-YP, as a vector alone control.

N-terminal GST-tagged constructs were generated by inserting *DED1, SSB2* and *TEF1*, amplified by PCR from genomic DNA, into pGEX-6p-1 (GE Healthcare) using restriction sites added during PCR to generate pGEX-6p-DED1, pGEX-6p-SSB2 and pGEX-6p-TEF1. Constructs were verified by restriction digest sequencing analyses prior to protein expression and purification. Mutant *TEF1* constructs were generated either by subcloning from mutant constructs obtained from Dr. Terri Goss Kinzy (UMDNJ; [7], [8]) or by site-directed mutagenesis using pGEX-6p-TEF1 as a template. Constructs used in β-galactosidase experiments have been previously reported [9], [10].

### B. Yeast strains and growth conditions

Yeast strains: BY4741(MATa, his3-1, leu2-0, met15-0, ura3-0) and isogenic eIF2A knock-out strain 4684 (MATa, his3-1, leu2-0, met15-0, ura3-0, *ygr054::KanMX)* were used in the investigation. To generate the C-terminal eIF2A-HA yeast strains used throughout this study, BY4741 yeast were transformed with PCR amplified fragments from pFa6a-3HA-KanMX6 [11] containing a 40 bp homologous sequence to areas within the eIF2A locus and selected on YPD containing 0.2 mg/ml geneticin, as described previously [12]. Yeast were propagated at 30^o^ C several days and large colonies were selected for PCR and Western blot screening of homologous integration of the HA-tag and resistance cassette. For β-galactosidase experiments, cells were cultured as previously described [6], [10].

Stress experiments were conducted by growing yeast transformed with YCplac111-YP in minimal selectable medium at 30°C until the OD_600_ reached 0.6. Cells were then treated with sorbitol by adding sorbitol to a final concentration of 1 M, with ethanol by adding 100% ethanol directly to the flask to a final concentration of 6%, or by addition of hydrogen peroixide to 0.32 mM and grown 1 h at 30°C prior to harvesting and processing. Cells examined for their response to heat shock with grown at 37°C for 1 h prior to harvesting.

### C. RT-PCR Analysis

Total RNA was extracted using the Masterpure RNA Purification Kit (Epicenter Biotechnology), and RT-PCR was performed using a one-step procedure as previously described [10]. Briefly, primers specific to the HA-eIF2A coding region were used in a multiplex experiment in combination with *PGK1-*specific primers as an internal control, as previously demonstrated [10].

### D. Sucrose Gradient Fractionation

YCplac111-YP transformed ΔeIF2A (BY4684) yeast were grown to log phase, and treated 10 min. with 10 µg/ml cycloheximide before harvesting cells. Cells were pelleted, washed with deionized water and resuspended in PB buffer (100 mM KCl, 2 mM magnesium acetate, 20 mM HEPES and 14.4 mM β-mercaptoethanol) containing a protease inhibitor cocktail tablet (Roche). Lysates were then prepared using glass bead lysis. Cleared lysates were generated and loaded onto 5–50% sucrose gradients that were fractionated with an ISCO Foxy Jr. gradient fractionator.

### E. 2D Gel Electrophoresis

Yeast lysates used in 2D gel analyses were prepared as follows. YCplac111-YP transformed ΔeIF2A (BY4684) yeast were sedimented, washed with sterile deionized water and resuspended in PB buffer supplemented with 0.25 mM Na_3_VO_4_, 10 nM NaF, 0.1 mM EGTA, 1 nM okadaic acid potassium salt and protease inhibitor cocktail tablet. Yeast were then lysed by glass bead disruption, and insoluble material was precipitated. The supernatant was extracted and mixed with two-dimensional gel loading buffer containing appropriate ampholytes and used in the isoelectric focusing dimension of 2D-gel electrophoresis.

### F. Western Blotting

For analysis of eIF2A protein turnover and Western blotting of 2D gels, lysates were prepared as described under *Sucrose Gradient Fractionation* or *2D Gel Electrophoresis*, respectively. After SDS-PAGE analysis, standard transfer procedures were employed for Western blotting of 2D gels. Antibody concentrations used were as follows: 1∶2000 α-HA (Cell signaling) or 1∶5000 α-Pgk1p primary antibody (Cell Signaling).

For blotting eIF2A from 2D gels derived from sucrose gradient experiments, fractions were precipitated overnight in 10% trichloroacetic acid, insoluble material was washed and resuspended in two-dimensional gel loading buffer containing the appropriate ampholytes. 2D electrophoresis was conducted as described in *2D Gel Electrophoresis*.

For analysis of eIF2A interacting partners, Western blotting was conducted according to standard procedures. Membrane-bound eIF2A-HA protein was detected after SDS-PAGE using a 1∶1000 dilution of α-HA-horseradish peroxidase-conjugated antibody (α-HA-HRP; Roche). Western blot detection of eEF1A protein was conducted using rabbit α-eEF1A antiserum (kindly provided by Dr. Terri Goss Kinzy at UMDNJ) at 1∶5000 dilution. Membranes from Kem1p immunoprecipitation experiments were probed with either 1∶2000 of α-Kem1p antiserum (kindly provided by Dr. Arlen Johnson at the University of Texas, Austin) or 1∶1000 α-HA-HRP antibodies.

### G. Affinity Purification

For identification of eIF2A-interacting proteins, YCplac111-YP transformed ΔeIF2A (BY4684) yeast were grown to log phase before harvesting in NETK Buffer (20 mM Tris-HCl, pH8.0, 100 mM KCl, 1 mM EDTA, 10% glycerol, 1 mM DTT, and 0.1% NP-40) containing protease inhibitors (1 µg/ml pepstatin, 0.2 mM phenylmethylsulphonyl floride, and 10 µg/ml of each aproptinin and leupeptin). Lysates were prepared by glass bead disruption. The supernatant was precleared with NETK-equilibrated protein A Sepharose 4 fastflow (GE Healthcare) before quantifying the protein concentration using a Bradford assay. The cleared lysate was then incubated with EZview red α-HA affinity matrix (Sigma) overnight at 4°C. Subsequently, the α-HA affinity matrix was washed with NETK buffer, and HA-tagged eIF2A protein was eluted twice from the α-HA affinity matrix by incubating for 1 h at room temperature in 0.2 mg/ml HA peptide in radioimmunoprecipitation assay buffer (1X phosphate-buffered saline, 1% NP-40, 0.5% sodium deoxycholate, and 0.1% SDS). The protein obtained in each eluate was precipitated by trichloroacetic acid precipitation prior to SDS-PAGE. Bands specific to the reactions containing HA-eIF2A protein were subjected to trypsin digestion and LC-tandem mass spectrometry as described under *Mass Spectrometry*.

GST-fusion proteins were prepared as described previously [13]. Briefly, BL21(DE3) cells (Stratagene) transformed with the appropriate pGEX-6p vectors were propagated, lysates were prepared according to standard procedures, and GST-fusion protein-conjugated beads were prepared using glutathione-sepharose 4B (GE Healthcare). The GST-pull-down reactions were performed as previously described [13]. Briefly, 140 µg total protein (from either eIF2A-HA yeast or C-terminal eIF2A-HA deletion yeast cell lysates) was pre-cleared with protein A Sepharose 4 fastflow before incubating with GST protein-conjugated glutathione-sepharose. Reactions were washed with NETK buffer and eluted with 2×Laemmli sample buffer followed by SDS-PAGE. Experiments examining the RNA-dependence of interactions were done as described above, except 2×reactions were conducted and split in two so one half could be treated with 10 µg RNaseA for 1 h at 4°C before analysis by SDS-PAGE and Western blotting.

For endogenous pulldown experiments, lysate from eIF2A-HA yeast was pre-cleared with protein A sepharose 4 fastflow resin. Subsequently, the supernatant was removed, and mouse monoclonal α-HA antibody (Sigma) or α-3-phosphoglycerate kinase (Invitrogen) was used to immunoprecipitate eIF2A-HA or 3-phosphoglycerate kinase, respectively at 4°C. After this incubation period, NETK-pre-equilibrated protein A Sepharose resin was added to each sample and incubated for an additional 2 h at 4°C. The resin was then washed in NETK buffer and eluted with 2× Laemmli sample buffer before SDS-PAGE. Reciprocal pulldown experiments of eEF1A were conducted with precleared eIF2A-HA yeast lysate as described above. Lysates were incubated with either α-eEF1A antiserum or preimmune serum provided by Dr. Terri Goss Kinzy (UMDNJ) before addition of protein A Sepharose 4 resin as described above. Reactions were then analyzed by SDS-PAGE and Western blotting.

Finally, pull-down experiments examining the interaction between Kem1p and eIF2A were conducted with eIF2A-HA yeast lysate as described for the endogenous eEF1A interaction studies.

### H. Mass Spectrometry

Mass spectrometry to identify proteins that interact with eIF2A was performed by the core facility at the Cleveland Clinic Foundation (analyzed by Dr. Mike Kinter and/or Dr. Belinda Willard). Briefly, gel slices were excised from the gel, reduced and alkylated and subjected to in-gel proteolytic digestion with trypsin overnight at room temperature. Peptides were then extracted with a solution of 50% acetonitrile and 5% formic acid prior to evaporation and resuspended in a final solution of 1% acetic acid. This solution was subjected to analysis with a Finnigan LCQ-Deca ion trap mass spectrometer system after peptide separation with a 10 cm×75 µm id Phenomenex Jupitor C18 reverse-phase capillary chromatography column.

## Results

**Figure 1:**
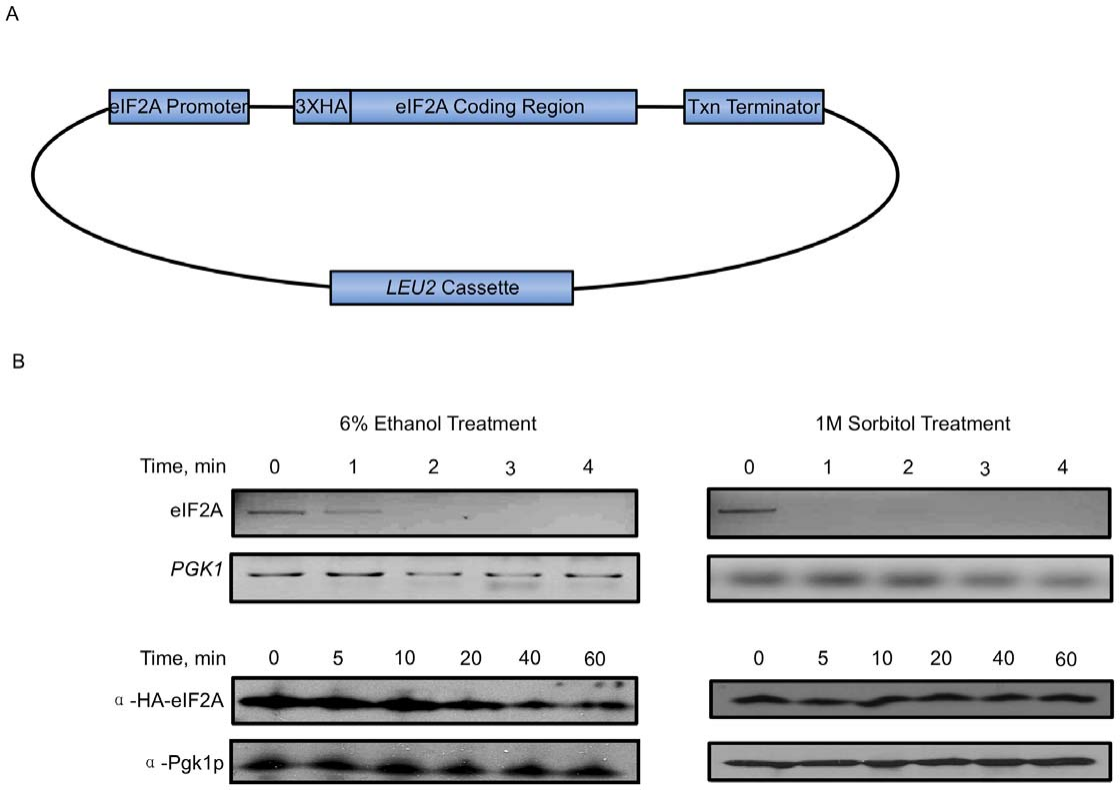
eIF2A mRNA and protein are turned over during stress conditions. *A.* Schematic diagram of the YCplac111-YP plasmid containing N-terminally HA-tagged eIF2A under the control of its native promoter. This plasmid contains a *LEU2* selection cassette and is used throughout the experiments presented in this manuscript, except where otherwise denoted. *B,* mRNA analysis (upper panels) and Western blot analysis of eIF2A and *PGK1* transcripts and protein levels (lower panels) indicating eIF2A mRNA is turned over during both 6% ethanol and 1M sorbitol stress in BY4684 ΔeIF2A yeast transformed with YCplac111-YP illustrated in *A*.

Since the role of eIF2A remains elusive in translational control, and it is known to repress IRES-mediated translation, we sought to delineate whether protein expression of eIF2A is reduced under cellular stress conditions that might result in enhanced internal initiation of translation. To evaluate eIF2A protein expression, we employed a construct in which the HA-tagged eIF2A coding region is positioned downstream of its natural promoter (Figure 1A). Unless otherwise stated, this construct is transformed into the ΔeIF2A/BY4684 yeast strain, as previously employed in our laboratory [5]. This experimental design is intended to reduce ambiguous results associated with overexpression and permit analysis of epitope-tagged eIF2A under transcriptional control mimicking conditions in wild type yeast strains.

With this expression system in place, eIF2A expression was analyzed under conditions of 6% ethanol treatment, heat shock, osmotic shock with 1M sorbitol, and oxidative stress with 0.32 mM H_2_O_2_, four stresses known to abrogate polysome formation. All four treatments resulted in rapid mRNA turnover, as eIF2A mRNA became undetectable by 2 min after application of the stress (Figure 1B). This is not a general observation because *PGK1* mRNA does not disappear over the time course of the experiment (Figure 1B). Interestingly, reduced mRNA expression only correlates with reduced expression of eIF2A protein under ethanol and heat shock stress conditions (Figure 1B), which is reduced to roughly 50% within the 30–60 min of treatment.

**Figure 2:**
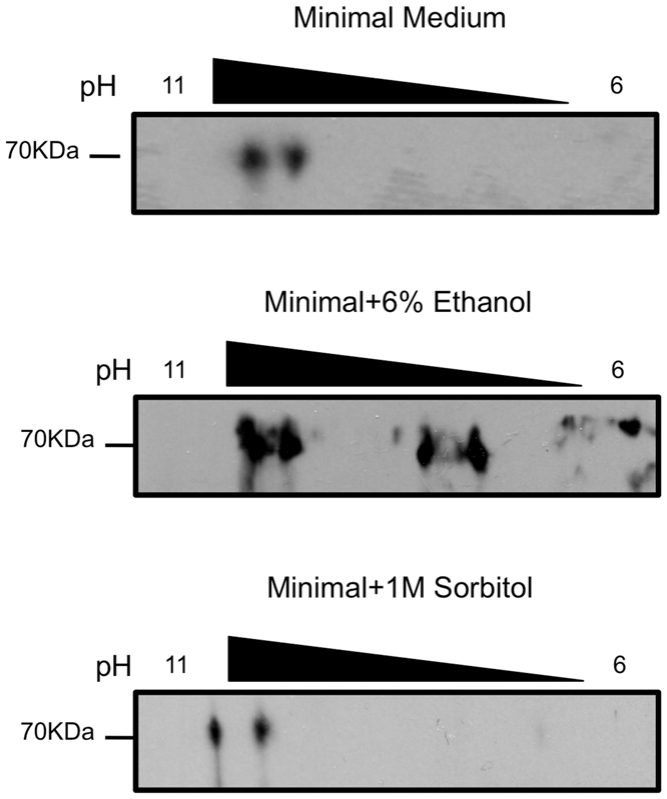
eIF2A is post-translationally modified during 6% ethanol treatment. 2D-gel electrophoresis followed by Western blotting for eIF2A from BY4684 ΔeIF2A yeast transformed with YCplac111-YP with or without the indicated stress.

Another potential mechanism to modulate protein function is to alter posttranslational modifications. We utilized 2D gel electrophoresis followed by Western blotting for HA-tagged eIF2A to assess changes in posttranslational modification of eIF2A. During unstressed conditions, eIF2A localizes to two spots in 2D electrophoresis, which reflects modification of a fraction of the eIF2A protein pool (Figure 2). Mass spectrometry analysis indicates that the modified form of the protein is phosphorylated at serine 564 (data not shown). When cells are grown in 6% ethanol (or heat shock, data not shown), eIF2A protein appears as four spots (Figure 2). Under sorbitol or H_2_O_2_ (data not shown) treatment, eIF2A protein appears to be the same two spots in 2D electrophoresis that resemble the spots observed during unstressed conditions (Figure 2). These results indicate that modification of eIF2A under 6% ethanol or heat shock treatment is specific for these two stress conditions. We have not checked a larger number of stress conditions, but anticipate that they will all yield a reduction in eIF2A mRNA levels and that some will result in reduced levels of eIF2A protein as well.

**Figure 3:**
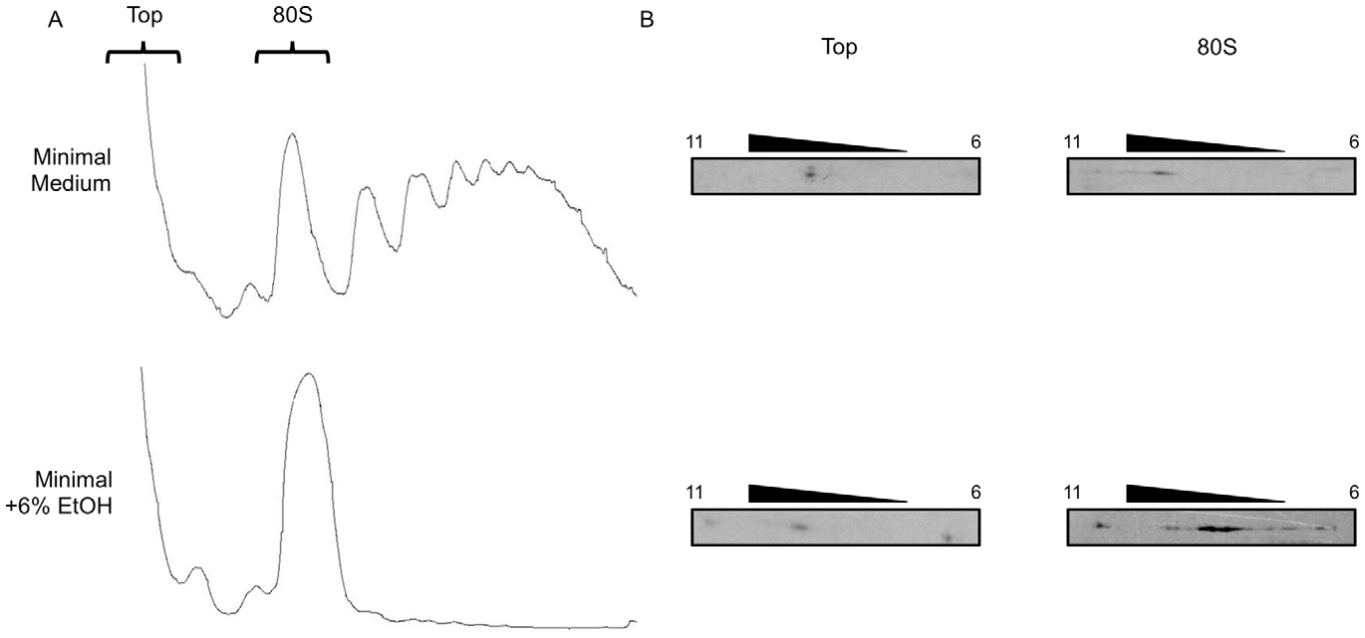
Post-translationally modified eIF2A appears in 80S preinitiation complexes during 6% ethanol stress. *A,* BY4684 ΔeIF2A yeast transformed with YCplac111-YP with or without 6% ethanol treatment were analyzed by polysome profiling. *B,* Subsequently, the top and 80S complex regions of the gradients (shown in *A*) were analyzed by 2D-gel electrophoresis and Western blotting with α-HA antibody to detect HA-eIF2A.

To assess whether the additional forms of eIF2A are functionally relevant, we examined distribution of eIF2A in polysome profiles (Figure 3). Previously, it was found that eIF2A localizes to 40S and 80S fractions [5]. For this reason we examined eIF2A species that exist in 80S complexes, and the top of the gradient (as potentially non-active controls) using 2D gel electrophoresis and Western blotting. This analysis indicates that under all conditions, HA-tagged eIF2A at the top of the sucrose gradient localizes around pH 9 (Figure 3B). Under normal growth conditions in which polysomes are intact, eIF2A in 80S complexes also localizes to a pH of 9. However, under 6% ethanol, but not sorbitol stress, in the 80S region there is a shift to the right in eIF2A localization in the 2D gels (Figure 3B; data not shown for sorbitol treatment).

**Figure 4:**
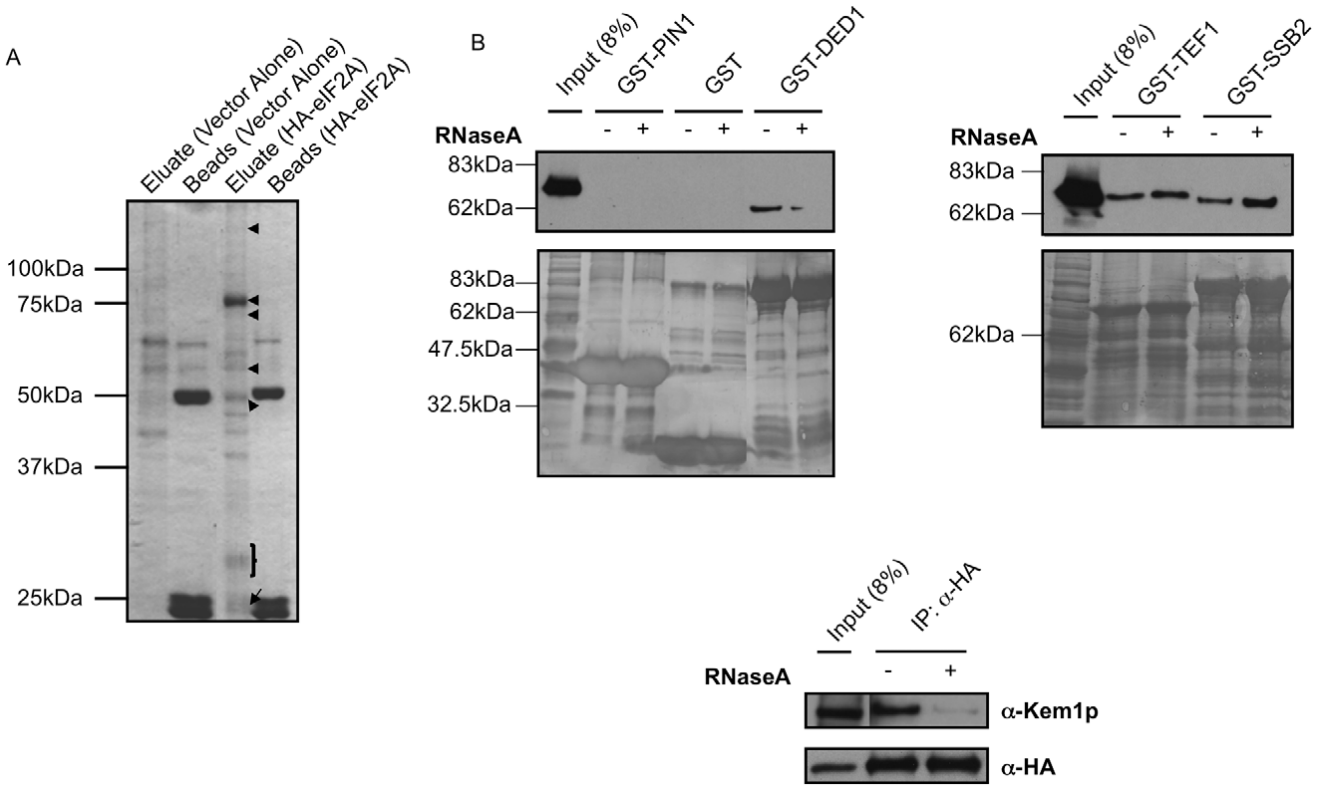
eIF2A-interacting partners identified by IP-mass spectrometry and independently confirmed. *A,* HA-eIF2A from lysate of BY4684 ΔeIF2A yeast transformed with either pTB328 vector alone or YCplac111-YP was immunoprecipitated with α-HA antibody. The immunoprecipitated protein complexes were analyzed by SDS-PAGE followed by mass spectrometry. Bands analyzed by mass spectrometry are highlighted by arrows or brackets. *B,* Some proteins identified in the IP-mass spectrometry experiment were analyzed for RNA dependence in either GST pulldown experiments (for Ded1p, Ssb2p and eEF1A/Tef1p) or immunoprecipitations followed by Western blotting (for Xrn1p/Kem1p, which cannot be expressed in bacteria) with lysates from eIF2A-HA yeast expressing a stable C-terminal HA-tagged eIF2A protein under the control of its endogenous promoter. Protein complexes were incubated in the absence (−) or presence (+) of RNase A followed by Western blotting for eIF2A-HA. In these experiments GST alone and GST-PIN1, a mammalian *cis*-*trans* prolyl isomerase, were analyzed as negative controls. Furthermore, controls for levels of GST-tagged proteins present in the pulldown reactions are shown by coomassie-staining of the membranes after Western blotting.

To delineate any interacting partners that might mediate translational control by eIF2A in addition to post-translational modification, we expressed HA-eIF2A (Figure 1A), and conducted immunoprecipitation followed by gel electrophoresis and mass spectrometry. Gel electrophoresis of the HA-eIF2A pulldown in parallel with the vector alone indicates that many proteins interact specifically with HA-eIF2A (Figure 4A). Bands highlighted in Figure 4A were excised and subjected to mass spectrometry. The major band in the HA-eIF2A eluate lane was positively identified as eIF2A, which corresponds to *YGR054w.* Most of the other proteins identified in this analysis are proteins that have been identified in translating mRNPs (many ribosomal proteins, eIF4A, Pab1p, Kem1p/Xrn1p, and eEF1A; Table 1). The striking number of translation components is consistent with a function for eIF2A in translation and previous findings documenting genetic and physical interactions with other translation components including: eIF5B, eIF4E, Rps11p, Rps11Ap and Rps13p [5], [14], [15].

**Table 1:** Proteins detected in IP-mass spectrometry experiments.

Gene Name	Common Name/Known Function	Percent coverage in LC-MS	Shown to interact previously	Reference
*PAB1*	Poly(A) Binding Protein	4%(2)	Negative Genetic	[35]
*TIF1*	Eukaryotic translation initiation factor 4A	9%(2)	Negative Genetic	[35]
*EFT1*	Eukaryotic translation elongation factor 2	3%(2)	ND	ND
*TEF1*	Eukaryotic translation elongation factor 1A	30%(20)	Positive Genetic	[35]
*KEM1*	XRN1, exoribonulease	4%(6)	Physical and Genetic	[14], [35], [36], [37]
*SSB2*	Ribosome-associated protein chaperone	27%(15)	ND	ND
*DED1*	ATP-dependent RNA helicase	8%(3)	Physical	[38]
*RPL15B*	Large ribosomal protein 15B	14%(2)	ND	ND
*RPS4A*	Small ribosomal protein 4A	18%(5)	ND	ND
*RPL3*	Large ribosomal protein 3	22%(10)	Physical	[14]
*RPS8A*	Small ribosomal protein 8A	14%(2)	Physical	[14]
*RPL2B*	Large ribosomal protein 2B	23%(4)	ND	ND
*RPL4B*	Large ribosomal protein 4B	30%(8)	ND	ND
*RPL7A*	Large ribosomal protein 7A	29%(5)	Positive Genetic	[39]
*RPL1B*	Large ribosomal protein 1B	22%(4)	ND	ND

HA-eIF2A protein complexes were immunoprecipitated and the protein complexes identified by mass spectrometry. Proteins detected by mass spectrometry that did not appear in the vector alone control are listed. ND under “Shown to previously interact” represents those proteins that have not been detected previously in the literature. NA under “Reference” indicates that no reference is applicable because it has not previously been detected.

To confirm some interactions between the predominant interacting proteins listed in Table 1 and eliminate RNA-dependent interactions resulting from immunoprecipitation of translating mRNPs (not direct protein∶protein interactions), GST pulldowns were conducted in the presence and absence of RNase A. Immobilized GST-Ded1p, eEF1A, Ssb2 and Kem1p/Xrn1p were incubated with lysate from a strain containing a chromosomally HA-tagged eIF2A under the control of the native eIF2A promoter (eIF2A-HA yeast). This strain is anticipated to express eIF2A at the normal physiologic levels of *Saccharomyces cerevisiae.* In this analysis, there were three classes of protein interaction results. The first class was the nonspecific controls including GST alone and GST-Pin1, a mammalian prolyl isomerase, which did not significantly interact with eIF2A. The second class of proteins interacted in a RNA-dependent manner, and included Ded1p and Xrn1p/Kem1p (Figure 4B). Finally, direct protein∶protein interactions with eIF2A were observed between both eEF1A and Ssb2p (Figure 4B). RT-PCR analysis with primers specific for 18S and 25S rRNAs indicates that the addition of RNase A yielded RNA that was extensively degraded during the course of the experiment (data not shown).

**Figure 5:**
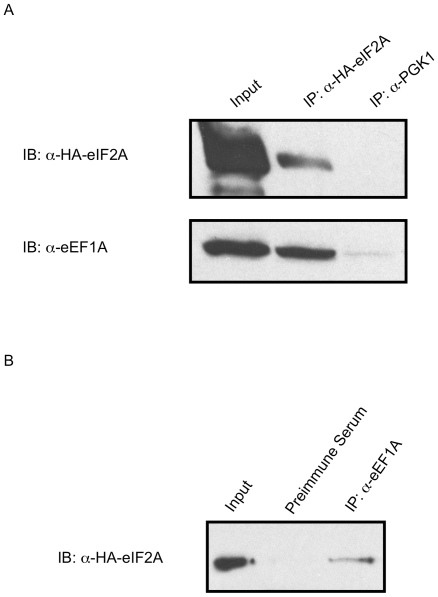
Endogenous verification of the interaction between eIF2A and eEF1A. *A,* Endogenous HA-tagged protein was immunoprecipitated from lysate of eIF2A-HA yeast. In these experiments, α-Pgk1p antibody was used as a negative control for the interaction. *B,* eIF2A-HA yeast lysates were used for endogenous immunoprecipitation of eEF1A protein in reciprocal reactions to those presented in *A*. Immunoprecipitations were analyzed for HA-tagged eIF2A and eEF1A protein by Western blotting analysis. Inputs refer to 1% of total protein added to each reaction in these experiments. Of note is that eEF1A is highly abundant within the cell, so even less apparent interactions are likely significant.

Since our laboratory had preliminary data that eEF1A interacts with the *URE2* IRES element, and it was shown to interact with eIF2A in a RNA-independent manner (Figure 4B), we attempted to validate the interaction between eIF2A and eEF1A under endogenous conditions. HA-eIF2A yeast were used to assess endogenous interaction between eIF2A and eEF1A. In this experiment, immunoprecipitation was conducted with either anti-HA or a nonspecific anti-Pgk1p control. eEF1A was only observed upon Western blotting in the presence of the anti-HA antibody (Figure 5A). A reciprocal pulldown using either anti-eEF1A immune serum and a preimmune serum nonspecific control also indicates interaction between eIF2A and eEF1A because HA-eIF2A only showed up in Western blotting with the anti-eEF1A immune serum (Figure 5B). In this experiment, the control blot for eEF1A is not shown because of overlap between the heavy chain and eEF1A signal.

**Figure 6:**
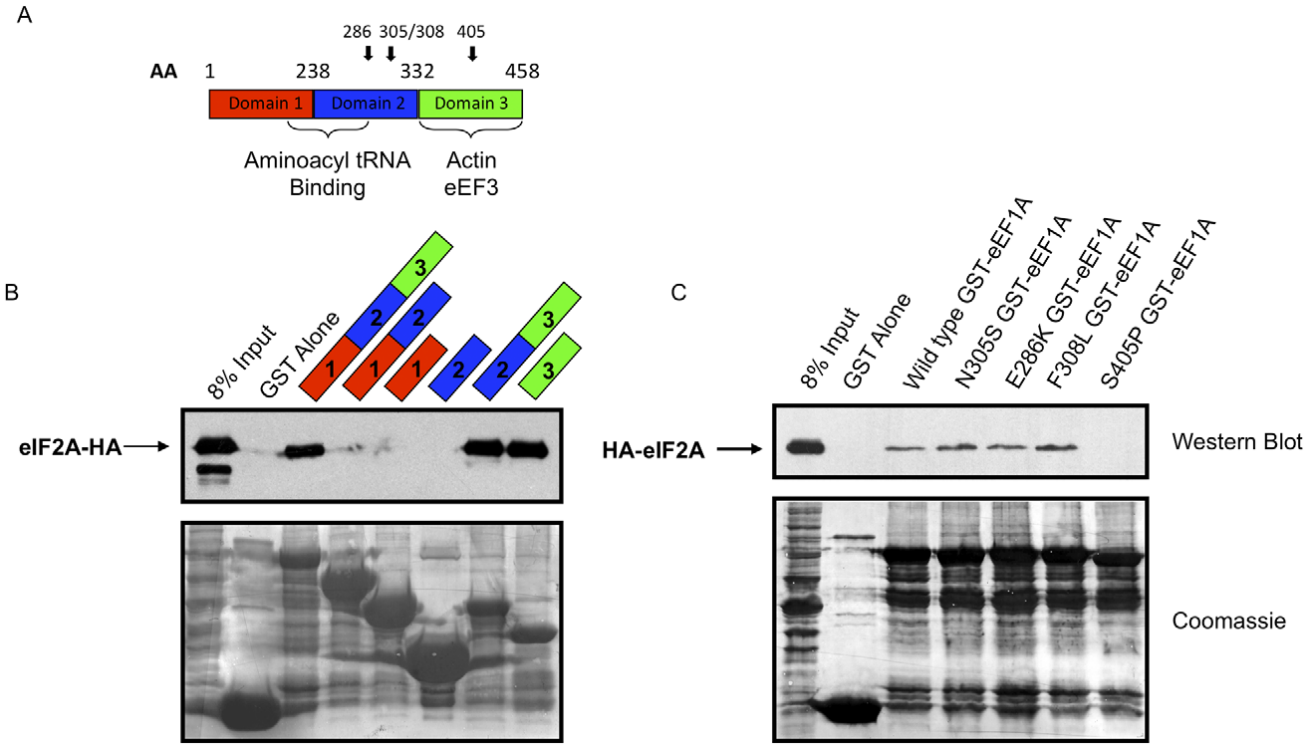
Domain 3 of eEF1A is required for interaction with eIF2A. *A,* The domain architecture of eEF1A is presented with amino acid demarcations at the boundaries of individual domains, and color coding of domains that is consistent throughout the figure. Bold arrows represent the location of mutations employed in *C*. Regions of eEF1A previously reported to be important for interaction with other proteins are highlighted with brackets. *B,* Domain deletions of eEF1A fused to GST were used to precipitate eIF2A-HA from eIF2A-HA yeast lysate. Western blotting for HA-tagged eIF2A was conducted on the precipitated material (upper panel). Domains of eEF1A present in the GST-fusion protein are indicated above the Western blot. GST fusion protein added to each GST-pulldown reaction was analyzed after Western blotting by coomassie staining of the membrane (lower panel). *C,* Domain 3 of eEF1A was found to be critical for the interaction between eEF1A and eIF2A. GST-fusion protein point mutants of eEF1A were used to confirm the necessity for domain 3 in the interaction between eIF2A and eEF1A using eIF2A-HA yeast. Each mutant is indicated in the figure. Western blotting followed by Coomassie staining of the membrane were conducted as described above (*B*).

Three domains exist within eEF1A that are responsible for different activities. The first domain is the GTP binding domain. Domain 2 is thought to interact with aminoacyl-tRNA, while domain 3 is important for actin binding and bundling [7], [16]. To identify the region of eEF1A that is important for the interaction with eIF2A, deletion mutants were produced as fusion proteins with GST and GST-pulldown experiments were conducted. Interestingly, HA-eIF2A was capable of interacting with eEF1A exclusively in the presence of eEF1A domain 3 (Figure 6A). Curiously, levels of input GST fusion proteins were lower in the pulldowns employing domain 3 as compared to other deletion mutants examined in these experiments (Figure 6A, bottom panel). Domain 3 of eEF1A was also shown to interact with eEF3 in *S. cerevisiae* and mediate tRNA delivery to the A-site of the ribosome [17]. To confirm the role of eEF1A domain 3 in the interaction, several point mutations were made that are known to abrogate the actin bundling properties of eEF1A [7], [8]. Of these mutations, only the S405P mutation in domain 3 resulted in reduced binding between eEF1A and eIF2A (Figure 6B). Since eEF1A is an essential protein, we were unsuccessful in determining differences in IRES-mediated translation in the sole presence of eEF1A mutants (which as the sole source of eEF1A yield slow growth phenotypes).

**Figure 7:**
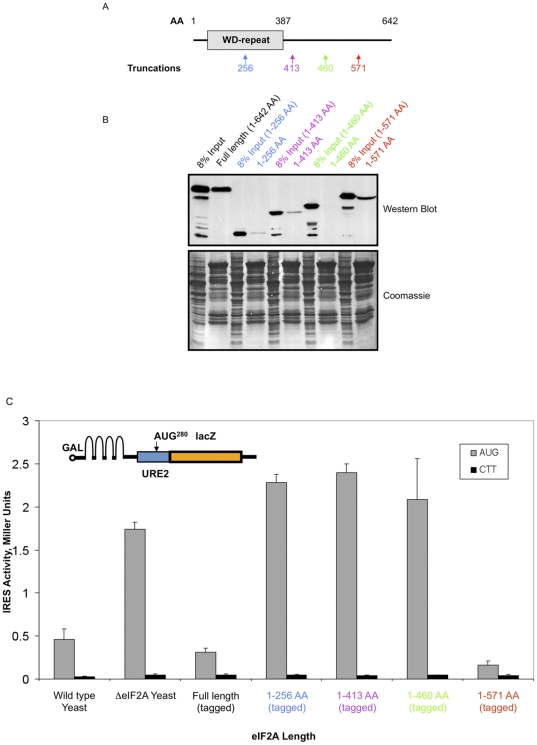
The C-terminus of eIF2A is important for interaction with eEF1A. *A,* A domain map of eIF2A, which indicates the presence of a WD-repeat that is predicted to fold into a seven-bladed β-propeller domain, indicates the amino acid size of yeast eIF2A and approximate location of the boundaries for the WD-repeat. HA-tagged C-terminal deletions of eIF2A were prepared, as described in *Results,* by homologous recombination. The location of each truncation is indicated below the schematic with the amino acid position and color coding that is used throughout the figure. *B,* Full-length GST-eEF1A was used in precipitation experiments with lysates from HA-tagged C-terminal eIF2A deletion yeast strains. Subsequent Western blotting for HA-tagged eIF2A truncations (upper panel; indicated with amino acid numbers) and coomassie staining (lower panel) of the reactions are indicated with 8% input controls for each reaction. *C,URE2* IRES function was analyzed in the presence of eIF2A truncations using the previously reported p281–4 plasmid shown in the inset [10], [18]. The p281–4 plasmid contains a galactose-inducible promoter, a stable hairpin that was shown to block cap-dependent scanning in yeast [18], and the *URE2* minimal IRES element upstream of the LacZ reporter sequence. Error bars indicate standard deviation from the mean. These experiments were repeated at least three times.

To identify which region of eIF2A is responsible for interaction with eEF1A, new yeast strains expressing C-terminally truncated, HA-tagged eIF2A were produced (Figure 7A). These strains expressed eIF2A under the control of its natural promoter as constructed for the HA-eIF2A yeast strain. Full-length GST immobilized eEF1A was utilized in experiments with lysates from these various yeast strains. Interestingly, levels of interaction between eEF1A and deletion mutants of eIF2A varied depending on the region of eIF2A that was deleted (Figure 7B). For example, while deletion of amino acids 571–642 resulted in a modest reduction of the interaction between eEF1A and eIF2A, deletion of amino acids 460–571 completely abolished the interaction (Figure 7B). Further deletions within eIF2A caused a recovery of the interaction between eEF1A and eIF2A. These results indicate that the region between 460–571 amino acids are critical for the interaction to occur.

To investigate a functional relevance for this interaction, we examined *URE2* IRES-mediated translation in eIF2A deletion strains. As a negative control for initiation, constructs in which the internal AUG codon were mutated to a CTT were used. Consistent with the interaction studies presented in Figure 7B, repression of internal initiation comparable to the wild type yeast strain was observed when the C-terminus of eIF2A was deleted (amino acids 571–642; Figure 7C). When amino acids 460–571 of eIF2A were deleted, the amino acids critical for the interaction between eIF2A and eEF1A, eIF2A-mediated repression of internal initiation is relieved to levels observed in the eIF2A deletion. These data are consistent with the interpretation that the eEF1A interaction is critical for initiation of the *URE2* IRES element and eIF2A-mediated repression, but may also reflect a loss of eIF2A function independent of its interaction with eEF1A.

**Figure 8:**
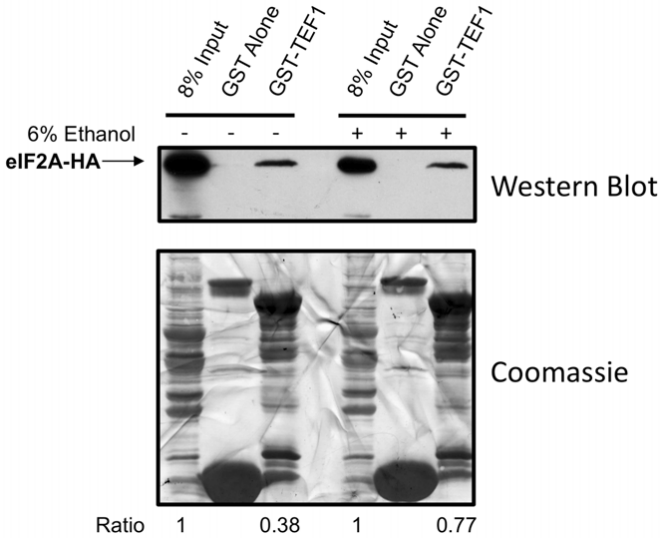
Ethanol treatment results in an increase in the proportion of eIF2A that interacts with eEF1A protein. BY4684 ΔeIF2A yeast transformed with YCplac111-YP (Figure 1A) were grown to log-phase growth and shifted to medium lacking (−) or containing 6% ethanol (+) for one hour prior to preparation of lysates. GST-pulldown reactions were then conducted with full-length eEF1A fusion protein and equivalent eIF2A-HA yeast protein lysate. Western blots for HA-tagged eIF2A (upper panel) and control coomassie staining (lower panel) of the resulting membrane are shown with 8% inputs for each lysate. Bands for input and HA-eIF2A protein precipitated were quantified using Image J software. Input band intensities were set to 1 and HA-eIF2A pulled down for each condition were normalized relative to respective input controls. The ratio of Input∶HA-eIF2A protein is indicated below each lane.

**Figure 9:**
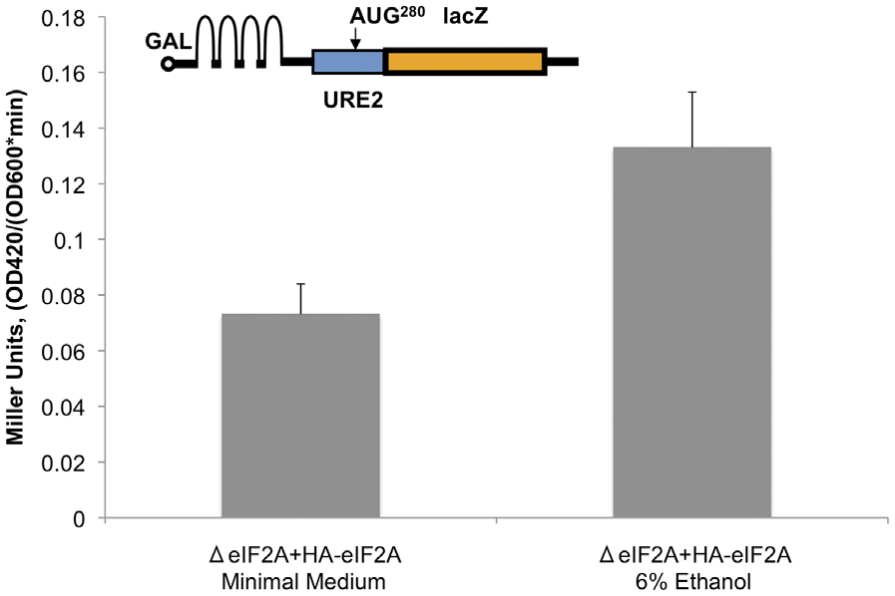
*URE2* IRES activity increases proportional to the increase in interaction between eIF2A and eEF1A. *URE2* IRES activity of the minimal IRES element (nucleotides 305–309) was evaluated as above (Figure 7C). This construct was transformed into ΔeIF2A yeast expressing HA-eIF2A from the YCplac111-YP plasmid (BY4684; ΔeIF2A+HA-eIF2A), and cultures were grown in the absence (minimal medium) or presence (6% ethanol) of 6% ethanol for one hour. Cells were harvested and β-galactosidase activity was measured. We have previously shown that there is no change in mRNA levels of this construct in an eIF2A-dependent manner [6], [10]. Error bars represent 15% of the mean, which is empirically acceptable for this type of experiment. These experiments were repeated several times.

To functionally characterize the interaction between eIF2A and eEF1A during ethanol stress, ΔeIF2A yeast transformed with YCplac111-YP were grown in either minimal yeast medium or 6% ethanol, and lysates were prepared for GST-pulldown analysis. GST pulldown experiments were conducted with eEF1A-conjugated glutathione-Sepharose followed by SDS-PAGE and Western blotting. The data from this experiment indicates that a larger percentage of eIF2A interacts with eEF1A during ethanol stress (Figure 8). If this interaction renders IRES-containing mRNAs more active in translation, internal initiation from the *URE2* IRES element would be expected to increase during 6% ethanol treatment. To measure *URE2* IRES-mediated translation, a construct harboring a stable stem loop upstream of the *URE2* IRES element to block cap-dependent translation was employed ([18]; Figure 9, inset). This construct contains a GAL1/10 promoter for tight regulation and inducible expression of the LacZ reporter, which is fused downstream of the minimal *URE2* IRES element. When this construct was transformed into ΔeIF2A yeast supplemented with HA-tagged eIF2A from YCplac111-YP and grown in the absence or presence of 6% ethanol for 1 h, IRES-mediated expression increased by 2-fold relative to control during 6% ethanol stress (Figure 9). Interestingly, the increase in *URE2* IRES translation correlates with a 2-fold increase in interaction between eIF2A and eEF1A (Figure 8).

## Discussion

Under normal conditions, yeast eIF2A is capable of suppressing translation from IRES-containing mRNAs, or at least for the three tested to date (*URE2*, *GIC1*, and *PAB1;* [5], [6]). eIF2A is also capable of directing binding of met-tRNA_i_ in an AUG-dependent (GTP-independent) manner, in contrast to the canonical GTP-dependent pathway (AUG-independent) directed by eIF2 [1]. From sucrose density gradient studies, eIF2A tends to be present on either 40S or 80S subunits [5]. These results indicate that eIF2A is working as a met-tRNA_i_ binding protein and that when it directs the binding of met-tRNA_i_ to 40S subunits, the rate at which it is converted to an 80S complex and then the subsequent release of eIF2A from the 80S ribosome is much slower than if eIF2 had directed the binding of the initiator met-tRNA. While this mechanism would explain how eIF2A suppresses IRES-mediated translation, it leaves open two major questions. If IRES-containing mRNAs encode stress response proteins, how do they overcome the eIF2A inhibition when stress occurs? Second, how is eIF2A specific for IRES-containing mRNAs since it has a relatively minor affect on m^7^G cap-dependent translation?

The answer to the first question is addressed in this study and appears to be two-fold. First, the level of eIF2A mRNA disappears very rapidly under a variety of stress conditions (this study and ref. 2) and remains decreased throughout the period of the stress. Thus, in the absence of mRNA, no additional eIF2A will be made during the period of stress thereby reducing translational suppression mediated by eIF2A. Secondly, eIF2A appears to be post-translationally modified during some stresses resulting in a more acidic form of the protein (the change in pI is equivalent to the addition of 2 phosphates, although currently we do not know the nature of the modification). This occurs in the case of ethanol treatment and may be responsible for the more rapid disappearance of eIF2A than when treated with sorbitol where eIF2A appears relatively stable. The conditions of ethanol treatment used within this manuscript mimic the diauxic shift in which cell growth is temporarily stagnant while protein expression is reprogrammed to deal with the ethanol stress [19], [20]. These data could suggest that the turnover of eIF2A is accelerated by the post-translational modification, and efforts to identify the type of modification and its location in eIF2A are in progress. Together, these data provide an explanation for elimination of IRES-mediated inhibition under ethanol stress conditions, but do not explain the stress response under sorbitol treatment.

As the eIF2A did not rapidly disappear in the sorbitol treated yeast even though polysome profiles show a dramatic reduction consistent with a shut off of translation initiation, might there be some advantage of having eIF2A remain present? Under these types of conditions, normally eIF2 activity is reduced (via phosphorylation) and eIF4F activity is also reduced (via the mTOR pathway). Although the reduction in eIF4F activity has “generically” been associated with the upregulation of IRES-mediated translation through the loss of the more competitive m^7^G cap-dependent pathway, a reduction in ternary complexes (eIF2•GTP•Met-tRNA_i_) would normally make it difficult to affect protein expression. In this light, there have been several publications on “eIF2-less initiation” which suggest that either eIF2A, eIF2D or eIF5B might be the protein directing the binding of the initiator tRNA [21], [22], [23], [24], [25], [26]. This possibility was examined using a constitutively active mutant of the eIF2 kinase GCN2 (Gcn2p_c_; [27], [28]). Using this mutant, we observed a roughly 40% decrease in m7G cap-dependent translation. At the same time, we noted that *URE2* IRES-mediated expression from the equivalent eIF2A knock out strain was 2 to 3-fold lower indicating that eIF2A appears to be substituting for the limiting ternary complex (data not shown). Thus, although eIF2A might function in the initiation pathway more slowly than eIF2, it still drives more expression under conditions equivalent to stress where there is extensive eIF2 phosphorylation. These results are consistent with the increased abundance of modified eIF2A protein present in 80S translation initiation complexes during 6% ethanol stress (Figure 3), which may act to more efficiently deliver met-tRNA_i_ for translation initiation of IRES-containing mRNAs. Indeed, *URE2* IRES activity is increased during ethanol stress by approximately 2-fold despite the disappearance of polysomes (Figure 3 and 9).

In the current literature, there has been rather extensive confusion about “eIF2A”. The primary source of confusion is with the alpha subunit of eIF2 reflecting the failure to distinguish between the lower case “a“(or α) and capital “A”. This can be readily confirmed by searching PubMed for just “eIF2A”. A second confusion relates to reports that eIF2, eIF2A, eIF2D or eIF5B can direct the binding of initiator tRNA to ribosomes [2], [3], [21], [22], [24], [25], [29], [30]. What is often missed is that the use of any of the proteins that are not eIF2 seems to only occur *in vivo* under conditions of eIF2 phosphorylation (of the alpha subunit, Ser51) when levels of ternary complexes are drastically reduced (and examination of polysome profiles indicates a collapse of the polysomes, as seen in Figure 3). Alternatively, from *in vitro* studies, the use of the different proteins is often influenced by the requirement for specific (and non-identical) templates to direct the binding of the initiator tRNA. These differences lead to the suggestion that it might be possible there exist even more such proteins, but that to date the correct template for their identification has yet to be identified. The only common feature for these proteins could be their participation in translation initiation events that are not the “standard” m^7^G cap-dependent initiation pathway.

The second question, how does eIF2A specially recognize IRES-containing mRNAs, is less well answered. Part of this recognition process may derive from the proteins that we have identified as interacting partners for eIF2A, most especially eEF1A and/or Ssb2p. In support of this possibility, a preliminary RNA pulldown experiment with the minimal *URE2* IRES element identified eEF1A as the predominant binding protein. Binding of eEF1A to a similar element, the BAT element, is consistent with this identification although it is noted that eEF1A is a basic protein and abundant in most cellular extracts [31]. The specificity in the eIF2A and eEF1A interaction is observed from the protein deletion studies, the studies with site-directed mutants of eEF1A revealing that the interaction is occurring via the C-terminal portions of both proteins and the fact that the interaction is not eliminated by addition of RNases. In direct assays for eIF2A activity, activity was quickly lost with deletion of any portion of the C-terminus of eIF2A past amino acid residue 460.

However, as predicted by the mfold algorithm [32], [33], neither the *GIC1* nor the *PAB1* mRNA appears to have a stem-loop IRES similar to that in the *URE2* mRNA. Thus, the possibility also exists that the utilization of eIF2A may reflect a decidedly different pathway than the canonical 80S initiation pathway. In particular, it might be possible that IRES-containing mRNAs are bound to the 40S subunit prior to binding of the initiator tRNA. From previous studies, it was shown with model systems that eIF2A required a template in order to bind the initiator tRNA to the 40S subunit while eIF2 did not [3]. And a similar observation has been made with respect to eIF2D and the HCV IRES (21). To date, most *in vitro* studies with IRES-containing mRNAs have assessed the translation factors required to obtain optimal yield of either 40S complexes or the toe print of a correctly positioned mRNA, but none have addressed the sequence in which any of the components were bound. If IRES-containing mRNAs uniquely bound to the 40S subunit first, this could provide the only explanation needed.

Even this does not rule out that eIF2A binding partners might influence its activity by influencing eIF2A's ability to bind to 40S subunits or by influencing its turnover. In this context it is noted that eEF1A has been characterized as an E3 ligase in the proteosome-directed degradation pathway and could perhaps serve a similar role in these studies [34]. In keeping with the E3 ligase specificity noted for eEF1A, eIF2A is N-terminally acetylated [4].

This work moves the field forward in providing mechanistic details on the role of eIF2A in the stress response and translation initiation of IRES-containing mRNAs. However, it is clear that considerably more work is required to fully understand either the biosynthetic pathway for the utilization of IRES-containing mRNAs or the role eIF2A may play in suppressing or supporting their translation. At the same time, the relevant role of non-eIF2 directed binding of the initiator tRNA also needs to be investigated to determine if this is restricted to any particular class of mRNAs, most especially IRES-containing mRNAs.
